# Effectiveness of integrated nutrition interventions on childhood stunting: a quasi-experimental evaluation design

**DOI:** 10.1186/s40795-021-00421-7

**Published:** 2021-05-13

**Authors:** Ester Elisaria, Jackline Mrema, Tariki Bogale, Giulia Segafredo, Charles Festo

**Affiliations:** 1 Ifakara Health Institute, Mikocheni, Keko Avenue. Dar es Salaam Branch, Dar es Salaam, Tanzania; 2 Children’s Investment Fund Foundation, ABC Place, 3rd floor Waiyaki Way, Westlands, Nairobi, Kenya; 3 Doctors with Africa CUAMM, Bagamoyo Road, Dar es Salaam, Tanzania

**Keywords:** Health education, Malnutrition, Stunting

## Abstract

**Background:**

Although malnutrition particularly stunting is recognized as multi-causal, there has been limited integrated nutrition interventions to reduce its burden in children under-fives and those existing are not well evaluated. This study tested the effectiveness of provision of health and nutrition education and promotion of home gardening in child stunting.

**Methods:**

The study used a quasi-experimental evaluation design. Two rounds of household surveys were done to assess changes in behaviors (uptake of Antenatal Care services and child feeding practices) and stunting among children under-5 years. The sample size was calculated to detect a 10% percent absolute baseline-to-end-line change in stunting. A two-stage stratified sampling process was used to sample 896 and 1736 households at each round of data collection in the intervention and control districts respectively. Mothers delivered in the past 24 months preceding the survey and all children under-5 years residing in selected households were eligible. The difference in difference (DID) analysis was used to estimate effect of the interventions. All ethical clearances were obtained from relevant authorities prior to data collection.

**Results:**

A total of 3467 and 4145 children under 5 years were recruited at baseline and endline respectively. The proportional of stunted children decreases from 35.9 to 34.2% in intervention and from 29.3 to 26.8% in the control sites. Overall, no statistically significant stunting reduction was observed between intervention and control sites. However, a significant effect was observed in intermediate outcomes; Uptake of iron folic acid (DID: 5.2%, (95% CI: 1.7–8.7), *p* = 0.003), health facility delivery (DID: 6.5%, (95% CI: 1.8–11.2), *p* = 0.006), pre-lacteal feeding (DID: − 5.9%, (95%CI: − 9.2, − 2.5), *p* = 0.001), breast feeding within 1 h after birth (DID: 7.8%, (95%CI: 2.2–13.4), *p* = 0.006) and exclusive breast feeding in children under 6 months (DID:20.3%, (95% CI: 10.5–30.1), *p* = 0.001).

**Conclusion:**

The 3 years program did not result in significant evidence of stunting reduction, but the observed effect on health and nutrition behavioural indicators are at the causal pathways to improved child nutritional outcomes in the long run. Implementation of these integrated packages over a longer duration is needed to witness significant reduction in the prevalence of stunting.

## Background

Although the world has observed positive progress in improvement of child and maternal health and nutrition indicators, levels of undernutrition, particularly stunting, continue to be high with approximately 149 million children under-five were stunted in 2018 [1]. African continent is by far the hardest hit by stunting with 30 countries out of 41 ranked worldwide with highest number of people experiencing more than one form of malnutrition (childhood stunting, anaemia in women of reproductive age and overweight among women) [2]. The consequences of stunting are profound including increased susceptibility to infections, mortality, reduced cognitive development, diminished educational attainment, less economic productivity in the later stage of life and lower birth weight of offspring [3]. There is also a close link between deprivation of food in early life and increased chances of adulthood chronic diseases [3]. The collective consequences of stunting cost up to 12% of the country Gross Domestic Product of developing countries [4].

Nearly 45% of all under-five deaths were attributed to malnutrition which translates to approximately 3.1 million deaths per year globally. Sub-optimal infant feeding alone contributes to 800,000 deaths per year and the prevalence of deaths was much higher in South Asia and sub-Saharan Africa than in other parts of the world [5]. Tanzania has made huge progress in reducing stunting in under-five children, from 43% in 1991 to 34% in 2015 [6]. However, disparities exists between regions with six regions (Ruvuma, Iringa, Rukwa, Kigoma, Njombe and Songwe) out of 26 having over 40% of stunted children [6]. This is unacceptably high by the WHO standards [7]. Several studies have linked poor nutritional status among pregnant women and women of reproductive age with adverse birth and nutritional outcomes among newborns and children [8, 9]. Evidence in Tanzania suggests that the prevalence of underweight (BMI < 18.5) among women of reproductive age has remained low and unchanged over the past 20 years. However, overweight and obesity (BMI > 18.5) has increased substantially. The 2015/2016 Demographic and Health Survey indicates, one in ten women aged 15–49 years were either underweight or obese and 18% were overweight [6]. Further, 45% of women of reproductive age and 57% of pregnant women were anemic [6].

Several studies focusing on implementation of integrated nutrition-specific interventions to reduce stunting yielded inconsistence findings [10–13]. In a controlled intervention study on complementary food supplements and dietary counseling on anemia and stunting, no impact on stunting among children 6–23 months was observed in China [14]. Another evaluation conducted in Ethiopia among children aged 6–36 months observed no improvement in stunting when an integrated approach (water, sanitation and hygiene (WASH), health and nutrition education) was implemented in a food-insecure population with very high stunting prevalence [15]. Haselow (2016) presented two studies implemented by Hellen Keller international in Baitadi and Kailali districts of Nepal and the Chittagong Hill Tracts in Bangladesh. In Baitadi, a cluster randomized control trial was used where communities were assigned to integrated interventions (Enhanced Homestead Food Production, Promotion of good nutrition and WASH), women’s empowerment, income generation and advocacy) or control. The study did not observe any impact on stunting. However, when a similar set of interventions was implemented in Kailali and Chittagong in a non-randomized control study, the Kailali district of Nepal revealed a 10.5% decline in stunting while the Chittagong Hill Tracts in Bangladesh achieved an 18% decline in stunting [13]. Methodological approach, packaging of intervention, duration of implementation and fidelity are some of the possible explanations for the observed variations.

### Description of the interventions

The government of Tanzania is aiming to eliminate stunting as a significant public health problem by 2030 [16]. As part of several initiatives, an integrated intervention program with the aim of reducing stunting in children under 5 years was implemented in Simiyu and Ruvuma regions of Mainland Tanzania from 2016 to 2019 by an Italian organization called Doctors with Africa CUAMM. The program targeted pregnant and lactating women and children underfive years and focused on provision of nutrition education and promotion of use of health services during the 1000-day window from conception to 2 years of child life. CUAMM local project team members, health care providers and Community Health care Workers (CHW) were main actors in the management and implementation program activities. The role of health care providers on program activities was to deliver the routine services (nutrition education, education on infant feeding, Iron and folic acid supplementation and management of Severe Acute Malnutrition (SAM) at the health facility which is beyond the scope of this paper.

Stunting screening, cooking demonstration, health and nutrition education were the core project activities done during the village health days. Since stunting is a chronic condition, it was screened twice a year. Cooking demonstration and community health education sessions were done quarterly (every 3 months) in each of the study village with each session lasting for half a day. The training materials known as *Mkoba wa Siku 1000* were adopted from the Ministry of Health, Community Development, Gender, Elderly and Children (MoHCDEC) and were used during facilitation of health education sessions. The training package had materials related to health education during pregnant and lactating, infant and young child feeding, handwashing, waste product management and birth preparedness.

The program also facilitated the formation of peer support group at village level with each having a maximum of 10 members and headed by community health care workers. The purpose of the groups was to facilitate provision of health and nutrition education among group members and the community at large and promote home gardening to ensure households availability of diversified food. The actual number of peer groups formed throughout the program implementation was not documented since this was not one of the core project implementation strategy. This paper generates an evidence from an evaluation work of these community program activities.

## Methods and design

The evaluation employed a quasi-experimental design to test the hypothesis that health and nutrition education and promotion of use of antenatal care (ANC) services in the first 1000 days of life will reduce stunting in children underfive years. Repeated household surveys were done in both control and interventions sites at baseline in June–September 2016, and endline in July–September 2019.

### Setting

The study was conducted in five regions of Tanzania mainland. Simiyu and Ruvuma regions were purposively selected as interventions sites by the implementers as they had a high prevalence of stunting, 33 and 44% respectively [6]. Simiyu is located in the Northern part of Tanzania and the South East of Lake Victoria while the Ruvuma is located in the Southern part of Tanzania. Four districts matched on health services availability, population, nutrition and mortality indicators were purposeful selected as controls. Uyui and Nzega districts located in Tabora region were paired with Simiyu region while Rufiji and Ruangwa districts in Coast and Lindi region respectively were paired with Ruvuma region. Districts with ongoing or planned similar nutrition program at the beginning of program activities were excluded from the controls sampling frame.

### Sample size and sampling

The sample size was calculated to detect a 10% percent absolute baseline-to-end-line change in stunting as a key indicator, using a baseline rate of stunting in the intervention and control districts of 40 and 26% respectively. Assuming a 15 and 5% absolute drop in stunting in intervention and control districts respectively over the 4-year period, a 5% type I error and 80% power, and an intervention to control ratio of 1:2, a total of 840 households in intervention districts and 1680 in control districts was dimmed sufficient after accounting for the 10% non-response. A two-stage cluster sampling process was used, with the first stage involving sampling of 56 villages, 28 from each site proportional to the district population size. This was followed by random sampling of 32 and 64 households from each village located in the intervention and control site respectively. All households with children under 2 years in the selected villages formed part of a sampling frame for the second stage sampling. Two and four households were purposeful added in the sample as replacement household in the intervention and control sites respectively. This resulted in interviewing more households than what was required due to mis-communication among team members in areas with poor phone network coverage. This has resulted in 13 and 49 more households being interviewed at baseline and endline survey from all study sites. These extra households were included in the final analysis.

### Study participants and eligibility

Mothers who delivered within the 24 months preceding the survey and all children under-5 years residing in the selected households were eligible. The 24 months were selected because child nutrition deteriorates in the period 6–24 months and after that age, the growth stagnates [17]. Infant feeding information was collected for all children under 24 months, as longer duration will introduce feeding pattern misclassification due to recall bias [18]. In household with two or more women with children less than 24 months of age, the one with the youngest child was interviewed to reduce recall bias.

### Measurement

#### Anthropometric measurements

Anthropometric measurements (weight and height) were taken from all under-five children who slept in the selected household a night preceding the survey and also to the blood parents of the children under-fives. Weight was assessed using the calibrated United Nations Children Funds (UNICEF) Electronic Scales [19]. The scale was placed on a flat surface, and children aged 2 years and above weighed while standing on a scale while those aged less than 2 years had their weight taken as a difference of weight of mother or caretakers with the child combined to that of the mothers alone. Weight was recorded to the nearest 0.1 kg. Height was measured using a height board. Children less than 2 years were measured lying on the length board while those aged 2 years and above were measured standing upright on the board. Height was recorded to the nearest 0.1 cm. Measurement procedures adhered to standardized protocols for anthropometry used in the construction of the international growth reference [19].

### Study questionnaires

A modified version of the validated Demographic and Health Survey (DHS) questionnaire was used during the interviews [6]. The tools captured i) mother’s demographic and reproductive history; ii) alcohol consumption and cigarette smoking history iii) history of health service utilization during ANC and the postnatal period, including Iron and Folic Acid supplementation, presumptive treatment for malaria and use of insecticide-treated bed nets during pregnancy and the postnatal period; iv) sources of breastfeeding information and a series of questions testing maternal knowledge on breastfeeding, complementary feeding and healthy eating during pregnancy and lactation; and v) infant feeding practices.

### Data management and analysis

Data was analyzed using STATA version 13 software. Descriptive statistics was done to establish the markers of household-level characteristics, health and nutrition indicators at each round of data collection. The anthropometry Z-scores were calculated using the WHO 2006 growth references [20]. The impact of a program was calculated using the difference in difference estimates with a *p*-value less than 0.05 at 95% level considered as significant. Principal components analysis was used to create a wealth index for each household based on the asset’s availability at household level.

### Quality assessment and control

All study tools were reviewed by relevant experts prior to data collection. Questionnaires were translated to Kiswahili and back-translated to English to ensure meaning was maintained. Field workers were trained for 4 days prior to each round of data collection to reduce within and between inter and intra-data variability. The training was followed by a day of field-testing of data collection tools. Weight and height/length measurements were standardized using FANTA guidelines [21]. All weighing scales were calibrated using an object of known weight prior to use. Data collection was done using electronic devices and uploaded to a central data server on daily basis allowing instant review and feedback to the field team.

### Ethical consideration

Ethical clearance from institutional (IHI/IRB/No:09–2016) and national (NIMR/HQ/R.8a/Vol.IX /2208) Research Ethics Committee were obtained. The study team paid courtesy visit to regional and district authority prior to beginning data collection activities. Permission letters were obtained and presented to village leaders during data collection activities. Before interviews, Research Assistants (RA’s) informed all study participants about the study objectives, risk and benefit of participating and study procedures. Only respondent who provided written consent were interviewed.

## Result

Overall, 2533 households were interviewed at baseline, and 2559 at end-line. Baseline and end-line population was characterized by a large family size (> 5). Closer to two third (> 64%) of the population had 5 or more family members. Teenage pregnancy ranged from 11.9 to 17.5% between baseline and end-line. Majority (> 79.1%) of the women interviewed were married. Most (> 63%) of the women had at least primary level education in both surveys. Generally, control sites had higher proportion of mothers with no education and poor socio-economic status compared to intervention sites at baseline and education continued to vary at end-line. Less than 7.4% of the mothers had BMI of < 18.5 **(**Table 1**).**

**Table 1:** Household and Socio-demographic characteristic of respondents the intervention and control sites, 2016 and 2019

	Baseline		End-line	
Indicator	Intervention N (%)	Control N (%)	Intervention N (%)	Control N (%)
**Number of Household**	853	1680	871	1688
**Household size**				
Median household size (range)	6 (2–30)	5 (2–30)	6 (2–28)	5 (2–27)
**Family size**				
2–4	277 (32.5)	604 (36.0)	267 (30.8)	564 (33.4)
5–7	344 (40.4)	663 (39.5)	369 (42.5)	686 (40.6)
8+	232 (27.1)	413 (24.6)	235 (27.0)	438 (26.0)
**Household wealth**				
1 (Poorest)	229 (26.9)	424 (25.3)	184 (21.1)	472 (28.0)
2 (Poor)	170 (19.9)	458 (27.3)	227 (26.1)	405 (24.0)
3 (Medium)	214 (25.1)	419 (25.0)	196 (22.5)	437 (25.9)
4 (Better off)	240 (28.1)	379 (22.6)	264 (30.3)	374 (22.2)
**Mother’s age (years)**				
15–19	149 (17.5)	252 (15.0)	104 (11.9)	240 (14.2)
20–29	453 (53.1)	911 (54.3)	491 (56.4)	883 (52.3)
30–39	208 (24.3)	446 (26.6)	237 (27.2)	472 (28.0)
40–49	43 (5.0)	70 (4.2)	39 (4.5)	93 (5.5)
**Marital status**				
Married	724 (84.9)	1328 (79.1)	760 (87.3)	1418 (84.0)
Single	81 (9.5)	226 (12.1)	65 (7.5)	174 (10.3)
Widow	8 (0.9)	12 (0.7)	7 (0.8)	4 (0.2)
Divorce	40 (4.7)	114 (6.8)	36 (4.1)	89 (5.3)
**Mother’s education**				
No schooling	135 (15.8)	432 (25.7)	114 (13.1)	439 (26.0)
Primary	588 (68.9)	1092 (65.0)	598 (68.7)	1062 (62.9)
Secondary +	130 (15.2)	155 (9.2)	159 (18.3)	187 (11.1)
^**a**^**Mother’s BMI**				
Thinness BMI < 18.5	52 (6.3)	103 (6.4)	40 (4.7)	121 (7.4)
Normal 18.5 < BMI < 25.0	624 (75.5)	1189 (73.3)	626 (74.1)	1132 (69.5)
Overweight ≥25.0	125 (15.1)	256 (15.8)	133 (15.7)	282 (17.3)
Obese ≥30.0	26 (3.1)	74 (4.6)	46 (5.4)	93 (5.7)

^a^ Pregnant women were excluded from the BMI calculations. The numbers in brackets are row percent unless stated

### Coverage of the interventions

Interviews with 2559 women were done at endline to assess the uptake of the intervention in both control and intervention sites. Out of these, 16.8% reported to ever attended at least one village health visit day in the intervention sites and 0.8% in the control. Of those who ever attended in the intervention sites, a half (51.4%) attended only once, and a quarter (24.7%) attended either twice or three times in a year. Forty percent of individual who ever attended village health visit day reported being trained on how to prepare food for children, and 17.8% on how to make a home garden and its benefits. Only 9% of interviewed women were members of peer support groups overall, 16.7% in the intervention and 4.6% in the control groups. A third, 31.5% (28.5–34.6) and 19.4% (17.7–21.5) of women reported having a vegetable garden at home in the intervention and control sites respectively.

### Nutritional outcomes of children

A total of 3467 and 4145 children under 5 years were recruited at baseline and endline respectively. Overall, there was no statistically significant difference in prevalence of stunting 3 years post the program implementation (DID: 0.8, 95%CI: − 3.4–5.1 and *p* = 0.704). However, the percentage of stunted children declined slightly from 35.9% (95% CI: 33.3–38.5) at baseline to 34.2% (95% CI: 31.9–36.6) at end-line in the intervention and from 29.3% (95% CI: 27.5–31.2) at baseline to 26.8% (95% CI: 25.2–28.6) at end-line in the control sites **(**Table 2**).**

**Table 2:** Nutritional indicators for children under-5 years between 2016 and 2019

Nutritional outcomes	Intervention		Control		Program effect (DID estimates)	
	Baseline	Endline	Baseline	Endline		
	Prev (%). (CI)	Prev (%). (CI)	Prev (%).(CI)	Prev (%). (CI)	% DID (95% CI)	***P*** value
	***N*** = 1334	***N*** = 1540	***N*** = 2333	***N*** = 2605		
***Stunting***						
Overall	35.9 (33.3–38.5)	34.2 (31.9–36.6)	29.3 (27.5–31.2)	26.8 (25.2–28.6)	0.8 (−3.4–5.1)	0.704
Severe	11.3 (9.7–13.1)	11.6 (10.2–13.4)	7.7 (6.7–9.9)	7.1 (6.2–8.2)	1.0 (−1.6–3.6)	0.442

### Uptake of maternity care

Almost all women (98%) interviewed received antenatal care during their most recent pregnancy from a skilled attendant at least once. There was no evidence of change in the number of ANC visits and timing of first ANC visit as a result of the intervention. However, a significant increase was observed in uptake of iron and folic acid (DID: 5.2, 95%CI: 1.7–8.7, *p* = 0.003), delivery at health facility (DID: 6.5, 95%CI: 1.8–11.2, *p* = 0.006), pre-lacteal feeding (DID: -5.9, 95% CI: − 9.2- -2.5, *p* = 0.001), breastfeeding within 1 h (DID: 7.8, 95% CI: 2.2–13.4, p = 0.006) and exclusive breast feeding from birth to 6 months (DID:20.3, 95%CI: 10.5–30.1, *p* = 0.001) **(**Table 3**).**

**Table 3:** Maternity care uptake and breastfeeding practice among mothers interviewed in 2016 and 2019

Indicator of interest	Intervention		Control		Program effect (DID estimates)	***P***-value
	Baseline	Endline	Baseline	Endline	% diff (95% CI)	
	Prev (%). (CI)	Prev (%). (CI)	Prev (%). (CI	Prev (%). (CI)		
	***N*** = 853	***N*** = 871	***N*** = 1680	***N*** = 1688		
**Number of ANC visit**						
Ever attended	98.6 (97.5–99.2)	98.0 (96.9–98.8)	98.0 (97.2–98.6)	97.5 (96.6–98.2)	−0.1 (−1.7–1.6)	0.924
At least 4 visits	48.2 (44.8–51.5)	69.0 (65.8–72.0)	51.8 (49.4–54.1)	71.0 (68.8–73.1)	1.5 (−4.0–7.1)	0.593
**Gestation age at First ANC (Months)**						
0–3	21.7 (19.0–24.6)	42.2 (39.0–45.6)	23.4 (21.4–25.5)	43.0 (40.7–45.4)	0.9 (−4.4–6.3)	0.728
4–6	68.0 (64.8–71.0)	50.9 (47.5–54.2)	64.3 (61.9–66.5)	48.5 (46.1–50.9)	−1.4 (−7.0–4.3)	0.636
7–9	9.6 97.8–11.8)	6.2 (4.8–8.0)	11.2 (9.8–12.9)	7.4 (6.3–8.8)	0.4 (−2.9–3.7)	0.825
**Uses of Iron and folic acid**						
Ever took	87.6 (85.2–89.6)	95.2 (93.5–96.4)	87.9 (86.2–89.3)	90.2 (88.7–91.6)	5.2 (1.7–8.7)	0.003
Took for 90+	17.9 (15.3–20.9)	40.6 (37.3–44.0)	29.1 (26.9–31.5)	47.4 (44.8–49.9)	4.5 (−1.2–10.2)	0.118
**Delivered at HF**	70.1 (66.9–73.1)	81.7 (79.0–84.2)	78.6 (76.5–80.5)	83.7 (81.9–85.4)	6.5 (1.8–11.2)	0.006
**Given pre-lacteal feeds**	14.2 (11.2–16.7)	7.1 (5.6–9.0)	9.3 (8.0–10.8)	8.2 (7.0–9.6)	−5.9 (−9.2- ^−^2.5)	0.001
**Ever breastfed**	98.7 (97.7–99.3)	99.7 (98.9–99.9)	99.0 (98.4–99.4)	99.8 (99.4–99.9)	0.2 (−0.8–1.1)	0.728
**Breastfeeding within 1 h**	59.1 (55.8–62.4)	66.2 (63.0–69.3)	66.3 (63.9–68.5)	65.6 (63.3–67.8)	7.8 (2.2–13.4)	0.006
**EBF per age (months)**						
0–1	91.9 (83.7–97.1)	94.6 (87.4–97.8)	95.1 (89.9–97.7)	88.6 (82.5–92.7)	9.2 (0.8–19.1)	0.070
2–3	75.0 (65.0–82.9)	84.3 (74.6–90.8)	68.5 (60.4–75.6)	53.1 (44.9–61.2)	24.7 (7.6–41.9)	0.005
4–5	47.6 (38.1–57.3)	61.5 (51.2–70.8)	43.9 (36.9–51.1)	28.7 (22.3–36.0)	29.1 (12.3–45.9)	0.277
0–5	70.0 (64.3–75.1)	79.7 (74.5–84.1)	66.7 (62.3–70.8)	56.1 (51.6–60.5)	20.3 (10.5–30.1)	< 0.001

*EBF* Exclusive breastfeeding, *hr* hour, *ANC* Antenatal clinic

## Discussion

This study investigated the effectiveness of integrated intervention (nutrition education, promotion of use of health services and home gardening) in the first 1000 days of a child’s life on reduction of child stunting. The findings show a slight reduction in stunting 3 years post the program implementation in both intervention and control sites, though not statistically significant. Although both groups have shown a non-significant decrease in stunning, this decrease is more pronounced in control group. This could be due to the high use of ANC services (early ANC attendance, at least 4 ANC visit, Iron and folic acid, and health facility delivery (Table 3)).

The no significant reduction of stunting in the intervention sites were unexpected findings but could be explained by the shorter duration of program implementation as stunting is a chronic condition that requires a longer period to change and low coverage of interventions at the community. Results from similar integrated interventions implemented in Ethiopia and Mozambique [15, 22] also found no improvement in child stunting. A systematic review by Goudet et al. in 2019 exploring the effect of these similar intervention reported no or moderate effect of the interventions on stunting [23]. The literature shows high mobility, lack of social services, and high loss to follow-up as possible explanation for this kind of findings [23].

Despite the program showing no impact on child stunting, a significant improvement was observed in the uptake of iron and folic acid, health facility delivery, pre-lacteal feeding, initiation of breastfeeding within 1 h of birth, and exclusive breastfeeding in children aged below 6 months. These results are in the intermediate or distal causal pathways for children’s stunting [24] and may results stunting reduction in the long run.

Further, these behavioural indicators have been defined by WHO as essentials nutrition action and recommended in early child life for optimum health, growth, and neurodevelopment [17]. Supplementation of iron during pregnancy, initiation of breastfeeding, exclusive breastfeeding, and appropriate complementary feeding practices during 1000 days were found to have a significant effect with child stunting [25]. In-depth analysis of demographic and health surveys in many sub-Saharan African countries has indicated an improvements in uptake of maternity care reduces the prevalence of stunting [26]. Based on these pieces of evidence from other studies, there is an indication that, the observed changes in behaviour among pregnant women using health services as well as infant and changes in child feeding practises impacted by the program in intervention communities could contribute to the reduction of child stunting in the future.

There were a number of methodological limitations in this study, many of which were outside the control of the project. Information collected from the mothers/caregivers was based on recall over varying time periods, which may introduce memory bias, particularly for infant feeding practices and other retrospective data relying on mother’s memory of the past events. Further, problems with implementation might be a possible explanation of the low coverage of intervention especially due to high staff turnover within CUAMM project management team, early phasing out of project activities due to shortage of funding. It remains unclear about the quality of training and supervision offered to CHW and to the community. So, no effect of the program on stunting reduction should be interpreted with care.

## Conclusion

Even though the program did not show an evidence of stunting reduction, a significant effect was observed in nutrition behaviours, ameliorating the uptake of iron and folic acid, health facility delivery, pre-lacteal feeding, and breastfeeding practices. These are in the causal pathway in reducing child undernutrition. More intensive intervention that are implemented at longer duration might be needed to see the effect of these interventions.

## Data Availability

The study datasets are available from the corresponding author on request.

## Abbreviations

BMI: Body Mass Index
CI: Confidence interval
DID: Difference in difference
HAZ: Height-for-Age Z-score
TDHS: Tanzania Demographic and Health Survey
WHO: World Health Organization
